# Effect of aging on the marginal fit of milled and printed zirconia crowns: an in-vitro study

**DOI:** 10.1186/s12903-025-05542-0

**Published:** 2025-02-11

**Authors:** Mahmoud S. Elsayed, Ahmed Y. El-Kouedi, Tamer E. Shokry

**Affiliations:** Department of Fixed Prosthodontics Faculty of Dental Medicine, Al-Azhar University, Cairo, Egypt

**Keywords:** Additive manufacturing, Artificial accelerated aging, CAD-CAM, Zirconia, Marginal fit

## Abstract

**Background:**

The additive manufacturing is one of the promising methods for fabrication of zirconia based restorations: However, studies on the marginal fit of zirconia restorations fabricated with this technique are sparse. This in vitro study was to measure and compare the marginal fit of milled and printed zirconia based crowns.

**Methods:**

Twenty-two human premolars were prepared to receive zirconia crowns. Each tooth preparation was scanned by a laboratory scanner. Digitally designed crowns were divided into two groups (*n* = 11) according to the method of fabrication either via 3D with a commercial 3Y-TZP slurry or a 5-axis milling machine using a multilayer zirconia blank. The marginal gaps were measured before, after cementation, and after thermomechanical accelerated aging. Kolmogorov-Smirnov and Shapiro-Wilk tests were conducted. Mann-Whitney U test was used to compare between the two techniques. Friedman’s test was used to compare between marginal gap distances before, after cementation and after aging. Dunn’s test was used for pair-wise comparisons when Friedman’s test is significant. The significance level was set at *P* ≤ 0.05.

**Results:**

Marginal discrepancies between the groups showed significant variations. In comparison to milled, 3D printing demonstrated a statistically significant greater marginal gap distance before, after cementation, and after aging (*P* < 0.001, Effect size = 2.361), (*P* = 0.011, Effect size = 1.28), and (*P* = 0.014, Effect size = 1.234), per respective.

**Conclusion:**

Significant differences were found between the two technologies used for fabricating zirconia crowns. The least discrepancies values were found with the milling technique.

## Introduction

Due to increasing aesthetic demands, the need for more conservative treatment options, and concerns regarding potential toxicological and hypersensitivity reactions associated with certain metals, both patients and dental professionals are increasingly favoring restorations that are metal-free and closely mimic the natural color of teeth. Yttria-partially stabilized zirconia (Y-TZP) ceramics have gained widespread popularity in dental applications, offering excellent aesthetic properties, biocompatibility, and superior fracture strength and toughness when compared to other dental ceramics [1].

Core substructures can be fabricated and subsequently covered with glass ceramics using methods such as layering, pressing, or computer-aided design/computer-aided manufacturing (CAD/CAM). However, due to direct exposure to functional stresses, including chewing, clenching, and moisture, the veneer may weaken over time and become prone to cracking or chipping. To mitigate this issue, monolithic zirconia restorations with a fully anatomical contour have been introduced, eliminating the need for an additional veneer layer [2]. However, the inability to obtain sufficient translucency of monolithic zirconia restorations make them aesthetically inferior when compared to veneered restorations [3].

Monolithic zirconia restorations are widely used in modern dentistry due to their biocompatibility, strength, and improved aesthetic qualities [4, 5]. Subtractive milling of zirconia restorations offers several advantages, including the integration of novel materials, reduced labor requirements, increased cost-effectiveness, and superior quality control when compared to traditional dental laboratory processes [5, 6]. However, this technology is associated with several manufacturing limitations, including substantial material waste from unused portions of the milled block, the necessity to replace milling tools after a set number of cycles, limited capacity to replicate surface morphology due to the dimensions of the milling instruments and the machine’s axis, as well as the potential formation of microscopic cracks during the milling process of the ceramic material [7, 8].

To overcome these limitations, considerable efforts have been made to fabricate dental restorations using various 3D printing techniques, including selective laser sintering (SLS), selective laser melting (SLM), stereolithography (SLA), inkjet printing (IJP), fused deposition modeling (FDM), and digital light processing (DLP), among others [5, 9].

The Digital Light Processing (DLP) process involves digitally segmenting the CAD file containing the desired design into extremely fine layers. DLP is employed to selectively place and cure photosensitive slurries, where the polymer network acts as a binder for the ceramic particles. Each layer is cured simultaneously using a projected light source. The resulting composites consist of distinct ceramic particles bound by the polymerized binder, forming the final green bodies. Excess or uncured material is then removed from these green bodies using compressed air and appropriate cleaning solutions. And then, the components are sintered to achieve their final properties after the binder is removed [10].

The marginal gap between the restoration and the prepared tooth structure is a key determinant of the longevity and success of a dental restoration. A marginal gap is defined as the vertical distance between the finish line of the preparation and the cervical margin of the restoration. A smaller gap reduces the exposure of the luting material to oral conditions, thereby decreasing the risk of pulpal inflammation, microleakage, cement dissolution, and periodontal tissue inflammation [11, 12]. The maximum marginal gap considered clinically acceptable has been reported to be 120 μm; however, a standardized criterion for clinically acceptable marginal gap has not yet been established [13]. Molin et al. [14]also indicated that marginal gaps ranging from 50 to 100 μm are deemed suitable for ensuring the success of restorations.

Artificial accelerated aging is a laboratory technique employed to predict the relative durability of materials when exposed to the challenging conditions of the oral environment. This method simulates the environment that restorative materials are expected to encounter in the oral cavity, thereby mimicking the potential environmental degradation they may experience [15].

Research on the marginal fit of zirconia crowns is crucial as it directly impacts the longevity, functionality and biological compatibility of restorations. Therefore, the aim of this in vitro study was to evaluate the marginal gap of zirconia crowns fabricated by additive manufacturing (3D printing) and subtractive (milling) technologies. The null hypothesis stated that there would be no significant difference in the marginal gap between 3D printed and milled zirconia crowns.

## Methods

The materials used in this study are listed in Table 1. Recently extracted human maxillary premolars were collected from the outpatient clinic of the Orthodontic Department at the Faculty of Dental Medicine, Al-Azhar University. The study proposal was submitted to the ethics committee of the college and was approved under reference number 600/1680.

**Table 1:** Materials used in the study

Material	Brand name	Manufacturer	Lot no
Bio Ceramic Zirconia slurry (3%Y-TZP)	INNI-CERA BCM-W500	AON, ZIPRO Dental, Korea	AI20630-016
Multi layered zirconia blank (3Y-TZP cervical, 5Y-TZP incisal)	NexxZr.T Multi	Sagemax, USA	YB4P8B
Glass Ionomer cement	BMS CEMBEST	BMS, Italy	122002-11

The average mesio-distal width of the selected teeth was 6.98 ± 1 mm, and the bucco-lingual width was 7.89 ± 1 mm. All teeth were free from cracks, cavities, or previous restorations.

### Sample size calculation

By adopting an alpha (α) level of 0.05 (5%), a beta (β) level of 0.20 (20%) i.e. power = 80%, and an effect size (d) of (1.26). Using a G*Power version 3.1.9.220, the sample size (*n* = 11) in each group was determined to be 80% to detect the difference between the means with a significance level of 0.05 [16]. The 22 premolars were randomly divided into two groups according to the method of fabrication of the zirconia crowns either by milling (group M) or 3D printing using a zirconia slurry (group P).

**Fig. 1:**
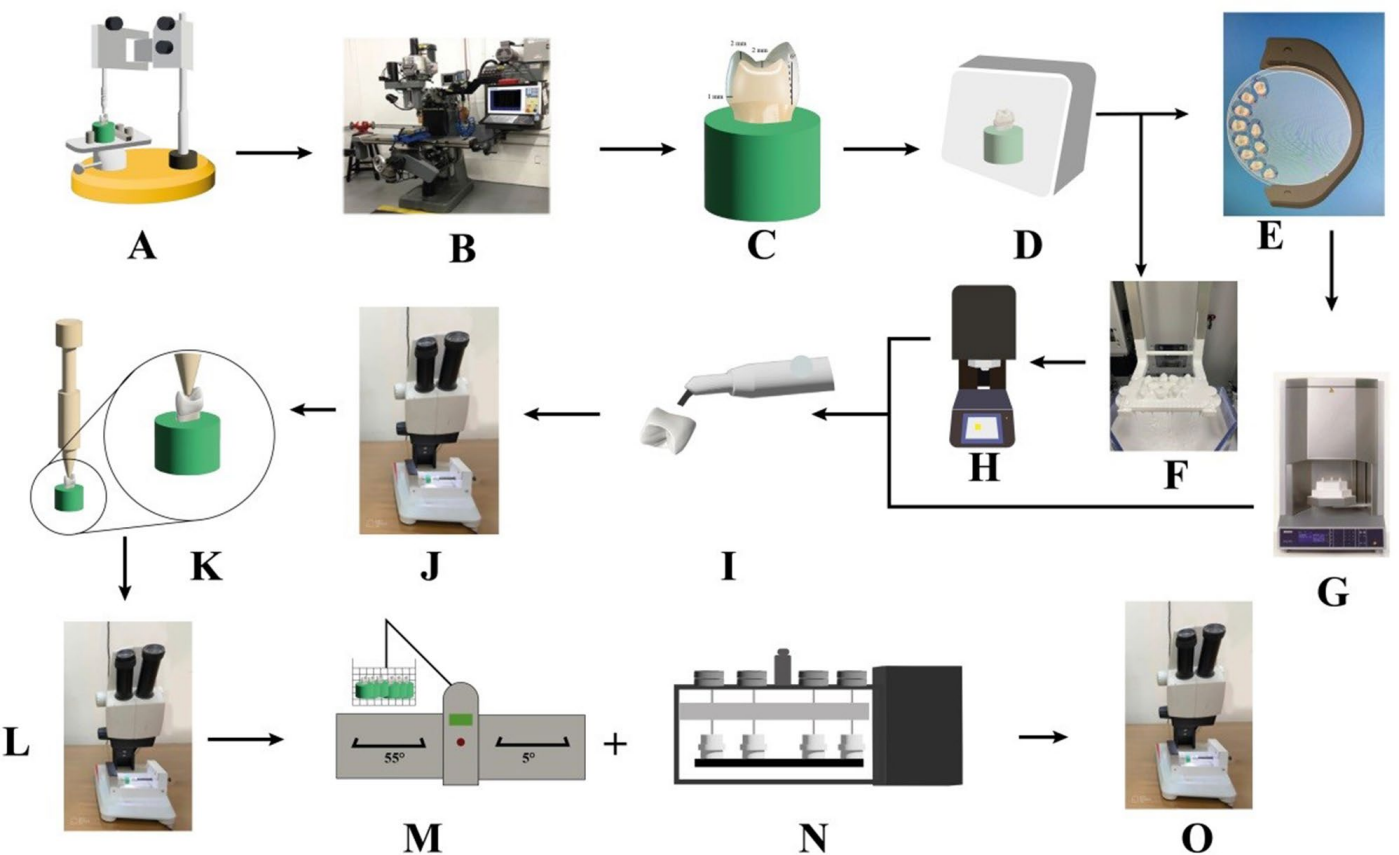
Schematic presentation of samples preparation and performed test in the study. **A**, Alignment of the long axis of the tooth to the long axis of the plastic mold with parallelometer. **B**, Reduction of all teeth by CNC milling machine. **C**, prepared tooth. **D**, Optical impressions were taken by Scanning and digitizing the prepared teeth using the 3 Shape D700L dental lab scanner. **E**, Milling process represented by nesting the designs in the blank for milling. **F**, Printing process represented by the printed samples on the printer platform. **G**, Sintering of milled samples. **H**, Sintering of 3d printed samples. **I**, Sandblasting of all samples. **J**, marginal gap measurement under stereomicroscope. **K**, cementation under static load 7 kg using loading device. **L**, marginal gap measurement after cementation. **M**, Thermocycling. **N**, mechanical aging in chewing simulator. **O**, marginal gap measurement again

A schematic presentation of the study steps is presented in Fig. 1. A parallometer (BEGO, Paraflex, Bremen, Germany) was used for proper orientation and vertical centralization of teeth inside the polyvinyl chloride (PVC) mold.

### Teeth preparation

To ensure consistent and standardized preparation dimensions, a Computer Numerical Control (CNC) machine, specifically the Premium 4820 (imes-icore, Eiterfeld, Germany), was employed. All tooth preparations were performed using this four-axis CNC milling machine, with a diamond endmill and an oily water coolant to facilitate the process.

**Fig. 2:**
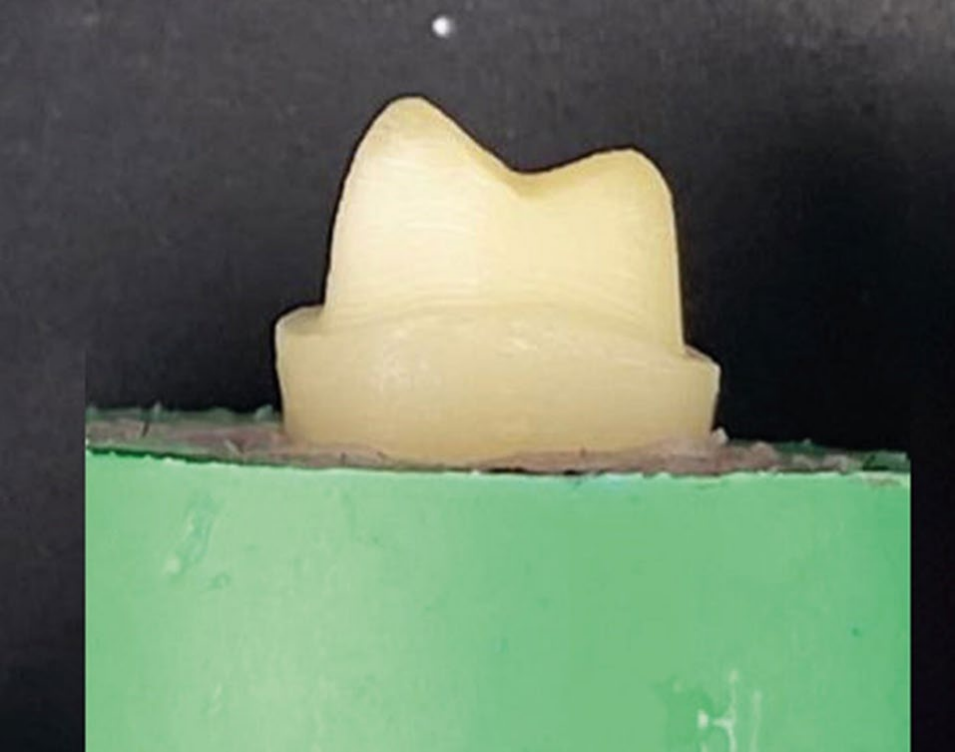
Tooth after preparation

The CNC machine was adjusted to reduce all teeth to satisfy the criteria of all-ceramic crown design (1-mm rounded shoulder finish line above cementoenamel junction (CEJ) by 1-mm, with 2.0 mm occlusal surface clearance and 6-degrees of axial convergence angle) [17] Fig. 2.

### Scanning and designing the crowns

Each prepared tooth was individually scanned using an extra-oral scanner (3Shape A/S, Holmens Kanal 7, 1060 Copenhagen, Denmark), resulting in a total of 22 STL files, which were then used to design 22 crowns. During the designing phase a biogeneric type was selected in the software (D700L scanner Dental System software version 2.17.3.0) and a spacer thickness of 60 μm was set and other parameters were calibrated according to the software default [17, 18]. The finalized design for each crown was stored as a standard triangulation tessellation language (STL) file to be used in both methods of fabrication.

### Crowns fabrication

Following the crown design process, the data were scaled up by 21.7% to account for sintering shrinkage before being transferred to the 5-axis milling machine (DWX-52 DI; DGSHAPE A Roland, Hamamatsu, Japan) to fabricate 11 crowns (group M) using the predetermined STL file. The enlarged crowns were milled from a presintered multilayer (3Y-TZP cervical, 5Y-TZP incisal) blank (Ø 98.5 mm, T16 mm). After milling the milling supports were cut and the crowns were sintered to its full density at a temperature of 1500 °C for 10 h using sintering furnace (TABEO-1/M/ZIRCON-100, MIHM-VOGT GmbH, Obrigheim, Germany).

The printed crowns (group P) were fabricated using a 3D printer (KUAIPRINT, Reddish Stone, Torino, Italy) for a commercial 3Y-TZP slurry employing Digital light processing technique (405 nm wavelength UltraViolet LED UV light and X/Y plane resolution of 50 μm pixels) considering the sintering shrinkage 31%. Following printing, the crowns were cleaned in an ultrasonic bath with a 50-vol% HDDA and 50-vol% ethanol mixture to get rid of any extra or uncured slurry [1].

The crowns were debinded and sintered in one step at 1500 °C for 21 h 45 m in a sintering furnace (Zotion F1, Chongqing Zotion Dentistry Technology Co., Ltd, Chongqing, China). All crowns manufactured by both techniques were sandblasted using alumina particles of 50 μm at 2 bar for 10 s [19] and glazed using (Ceramotion Zr Paste Glaze, Dentaurum, Germany) according to the manufacturer instructions.

### Marginal gap assessment

**Fig. 3:**
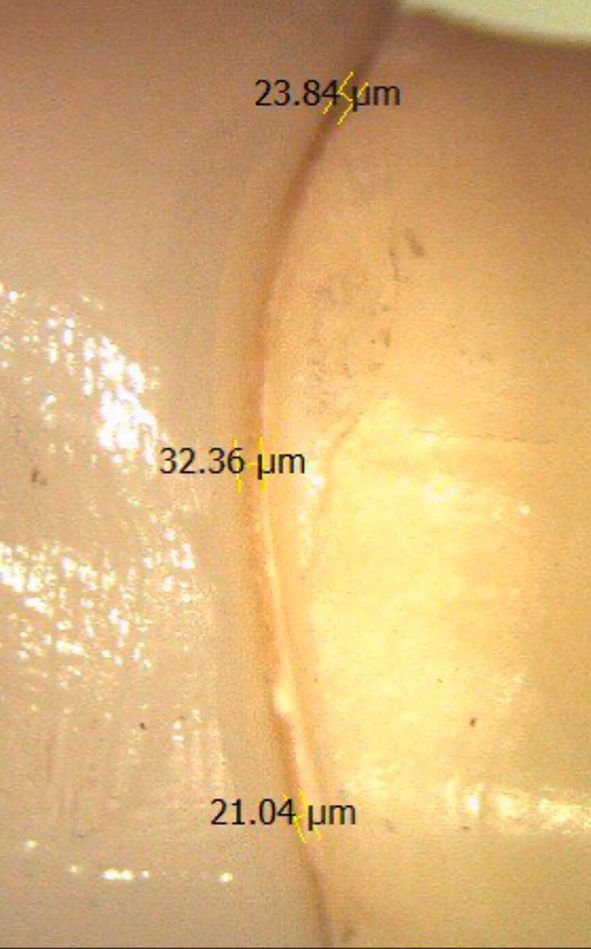
Screen shot of stereomicroscope measurement of marginal gap measurement before cementation

Each crown was checked for seating and placed onto its corresponding prepared tooth and stabilized using a sample positioning device. The vertical marginal gap before cementation was calculated by measuring the vertical distance between the crown margin and the finish line using an integrated digital camera and stereomicroscope device (Wild Lecia M8, Leica Mikrosysteme, Heerbrugg, Switzerland) with a 35X magnification and analysis software (Leica LAS AF LITE 4.10.0) Fig. 3.

**Fig. 4:**
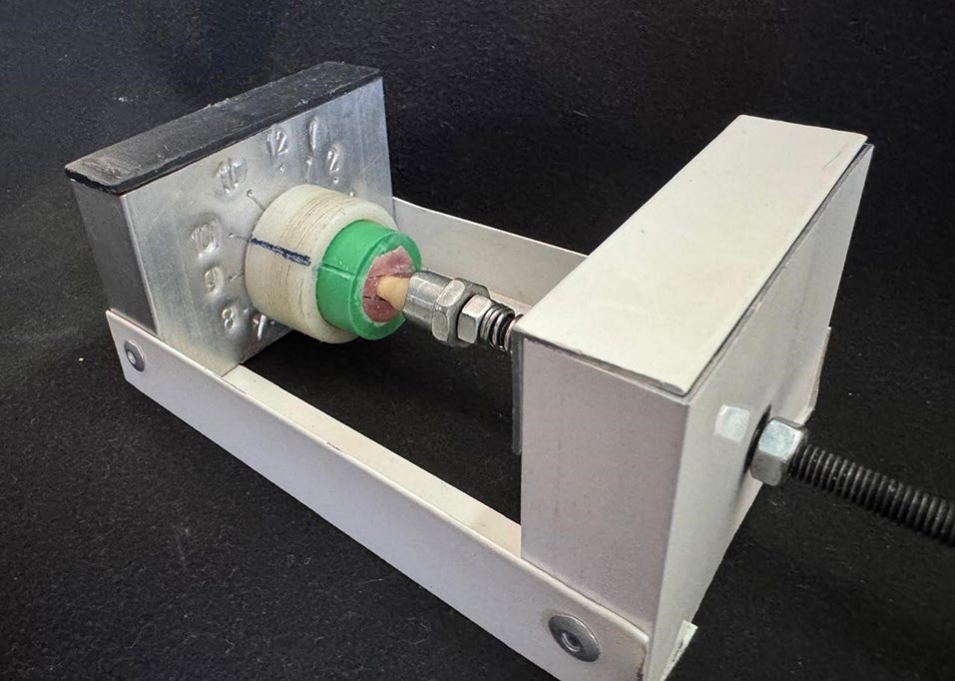
The sample positioning device (SPD)

A Sample Positioning Device (SPD), shown in Fig. 4, was designed to address the challenge of consistently measuring the marginal fit at the same distance and angle from the microscope capturing unit. This SPD is a straightforward tool consisting of a calibrated gauge, a fixed base, reference marks, and a coil spring to apply a static load during measurements. The samples were placed into the corresponding housing of the holding device, ensuring alignment of the groove previously created in the sample with a.

mark in the device. The housing, which holds the sample, can rotate over the fixed base and be locked at each of the 12 points for precise measurement. A total of 12 measurements were taken on each sample, with three measurements at predefined locations on each surface [17, 20].

### Crowns cementation and marginal gap assessment

**Fig. 5:**
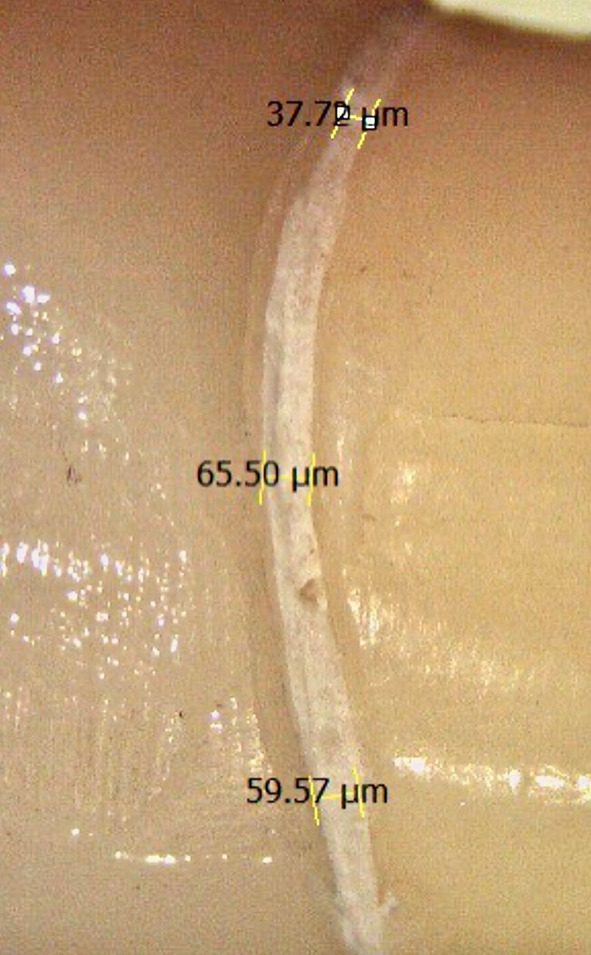
Screen shot of stereomicroscope measurement of marginal gap measurement after cementation

For the cementation process, the glass ionomer cement (BMS CEMBEST, BMS, Italy) was used for cementation under static load that produced a constant seating load of 7 kg (approx. 70 N) [21]. Excess semi-hard cement was removed using a manual curette, with gentle strokes applied from the crowns toward the teeth. The margins were then polished with rubber cups to achieve a smooth finish. The marginal gaps were reassessed again after cementation Fig. 5.

### Artificial accelerated aging

All samples were subjected to 5000 cycles thermal. Each water bath (Robota automated thermal cycle; BILGE, Turkey) had dwell times of 25 s and lag times of 10 s, with the low temperature 5ºC and 55ºC was the high temperature (which is clinically equivalent to six months) [22].

After the thermal aging, all samples were exposed to mechanical cyclic loading using the ROBOTA chewing simulator (ROBOTA chewing simulator (Model ACH-09075DC-T, Germany) for 75000 cycles, 50 N, and a range of frequency 1–1.6 Hz in a wet condition which resembles approximately six months under function according to previous studies [23].

### Marginal gap assessment after aging

Following the completion of cyclic loading, the vertical marginal gaps were recorded using a stereomicroscope at the same magnification with the same protocol. Collected data was tabulated and statistical analysis was performed.

## Results

Numerical data were explored for normality by checking the distribution of data and using tests of normality Kolmogorov-Smirnov and Shapiro-Wilk tests. All data showed a non-normal (non-parametric) distribution. Mann-Whitney U test was used to compare between the two techniques. Friedman’s test was used to compare marginal gap distances before, after cementation, and after aging. Dunn’s test was used for pair-wise comparisons when Friedman’s test was significant. The significance level was set at *P* ≤ 0.05. Statistical analysis was performed with IBM SPSS Statistics for Windows Version 23.0. Armonk, NY: IBM Corp.

### Comparison between fabrication techniques

Before, after cementation, as well as after aging; group **P** showed statistically significantly higher marginal gap distance than group **M** (*P*-value < 0.001, Effect size = 2.361), (*P*-value = 0.011, Effect size = 1.28) and (*P*-value = 0.014, Effect size = 1.234), respectively. Table 2.

**Table 2:** Descriptive statistics and results of Mann-Whitney U and Friedman’s tests for comparison between marginal gap distances (µm) of the two techniques

Measurement	3D printing (*n* = 11)	CAD-CAM (*n* = 11)	*P*_1_-value	Effect size (d)
Before cementation				
Median (Range)	37 (19–80)	17 (14–30)	< 0.001*	2.361
Mean (SD)	41 (16) ^B^	20 (6) ^B^		
After cementation				
Median (Range)	45 (15–76)	19 (11–55)	0.011*	1.28
Mean (SD)	42 (19) ^A^	22 (12) ^A^		
After aging				
Median (Range)	49 (17–77)	21 (16–58)	0.014*	1.234
Mean (SD)	45 (19) ^A^	25 (12) ^A^		
P_2_-value	0.035*	0.013*		
*Effect size (d)*	0.306	0.397		

*: Significant at *P* ≤ 0.05

P_1_-value: for Mann-Whitney U test

P_2_-value: for Friedman’s test

Different superscripts in the same column indicate statistically significant changes within technique

### Changes after cementation and after aging with each fabrication technique

There was a statistically significant change in marginal gap distance within each technique (*P*-value = 0.035, Effect size = 0.306) and (*P*-value = 0.013, Effect size = 0.397), respectively. Table 3.

**Table 3:** Descriptive statistics and results of Friedman’s test for comparison between marginal gap distances (µm) before, after cementation and after aging with each technique

Measurement	Group P (*n* = 11)	Group M (*n* = 11)	
**Before cementation**			
Median (Range)	37 (19–80) ^B^	17 (14–30) ^B^	
Mean (SD)	41 (16)	20 (6)	
**After cementation**			
Median (Range)	45 (15–76) ^A^	19 (11–55) ^A^	
Mean (SD)	42 (19)	22 (12)	
**After aging**			
Median (Range)	49 (17–77) ^A^	21 (16–58) ^A^	
Mean (SD)	45 (19)	25 (12)	
***P*** **-value**	0.035*	0.013*	
***Effect size (d)***	0.306	0.397	

*: Significant at *P* ≤ 0.05, Different superscripts in the same column indicate statistically significant changes within technique

Pair-wise comparisons revealed that there was a statistically significant increase in the mean marginal gap after cementation, followed by a non-statistically significant change in the mean marginal gap after aging. While the mean marginal gap after aging showed a statistically significantly higher value than before cementation.

## Discussion

This in vitro study examined the marginal gap of 3D printed and milled zirconia crowns. The null hypothesis that the marginal gap would not be influenced by the fabrication techniques was rejected.

The study was conducted on extracted human teeth, which were used as models to simulate ex vivo procedures enabling a controlled evaluation of variables being investigated [24]. A parallometer was used to mount the teeth in acrylic molds, ensuring standardization and minimizing preparation errors [25]. A CNC machine was employed to perform a standardized tooth preparation, thus eliminating the influence of preparation geometry and margin configuration on the adaptation of the crowns [26]. However, it should be noted that no milling machine is entirely free from variability. Even with a high-precision device, minor discrepancies in tooth preparation can occur due to the inherent limitations of the machine’s accuracy. Such minor variations may have a minimal impact on the overall marginal gap measurements. Nevertheless, as these discrepancies were consistent across all samples, their influence on the study outcomes is considered negligible. The 3Shape D700L scanner (3Shape A/S, Holmens Kanal 7, 1060 Copenhagen, Denmark) is a highly accurate desktop scanner commonly used in dental laboratories for creating digital impressions and designing dental restorations such as crowns. As stated by the manufacturer, the D700 series scanners achieve a scanning accuracy of 5 μm, validated under ISO 12,836 an international standard for evaluating the performance of dental scanners. Studies have confirmed the reliability of the D700L scanner in delivering precise digital models [27, 28], which ensures accurate crown designs in the context of this study.

Ayad [29] identified significant differences in marginal openings among various die materials when using a shoulder margin design, with greater marginal openings observed compared to those seen with light chamfer and rounded shoulder margin designs across both die materials. Based on these findings, this study employed a rounded shoulder finish line, which is compatible with non-metallic restorations and contributes to reducing stress and minimizing the risk of fracture.

Ensuring an adequate cement spacer is essential for achieving accurate restoration margins [18, 30, 31]. To minimize the effect of this variable on marginal discrepancies, all crowns in this study were fabricated with a 60 μm cement spacer, which was digitally set 1 mm coronal to the finish line [20].

This study evaluated marginal gap through direct observation with external measurements using a stereomicroscope set at a fixed magnification of 35X [5, 17]. This method offers the advantage of being non-invasive, accurate, and reproducible, making it suitable for determining the the marginal gap of the entire sample. However, it can be challenging to replicate measurements from the same angle and differentiate the true marginal gap from its projection. To address this issue and ensure measurement standardization, a specially designed sample positioning device (SPD) was used to secure the specimens in place, enabling repeated measurements from a consistent angle aligned with the microscope’s focal plane.

The marginal gap was measured at 12 points for each tooth three in each surface measurement error was reduced, and the restoration’s circumferential fit could be accurately estimated [17, 20].

Accelerated aging tests provide crucial data for predicting the lifespan of ceramic restorations. Given the high costs and time requirements of clinical trials, laboratory simulations are often used to replicate clinical conditions. Thermal cycling, a process that occurs in vivo, is commonly included in these simulations to mimic real-life conditions. In this study, a total of 5000 cycles were performed, which corresponds to approximately six months of clinical aging. Each water bath had a dwell time of 25 s and a lag time of 10 s. The lowest recorded temperature was 5 °C, and the highest recorded temperature was 55 °C [22].

The parameters for masticatory simulation were calibrated to physiological values documented in the literature [23]. The number of mechanical cycles in the reviewed studies varied significantly, ranging from 10,000 to 3.6 million cycles, equivalent to 5 months to 14.4 years of clinical service. In our study, we applied 75,000 cycles to represent approximately 6 months of functional clinical service [23].

Recent studies have explored the application of 3D-printed zirconia crowns in dentistry, focusing on their fit, precision, and trueness compared to milled counterparts, suggesting their potential viability in clinical settings [3, 32, 33].

The mean marginal gap values for all zirconia crowns produced using both techniques were found to be below 50 μm, indicating that all specimens were within the clinically acceptable range. The mean marginal gaps for the milled zirconia crowns (group M) before cementation, after cementation, and after artificial accelerated aging were 20 μm, 22 μm, and 25 μm, respectively. This result is consistent with several studies that have investigated the marginal gaps of monolithic zirconia crowns, which report a range from 18 to 120 μm for CAD/CAM restorations [34–38]. In comparison, the marginal gaps for the 3D printed crowns were 41 μm, 42 μm, and 45 μm, respectively.

The mean marginal gap of the milled crowns in this study was notably smaller compared to that of the 3D printed zirconia crowns. These results yyy [5, 39–42] However, this contradicts a previous studies that reported no significant difference with marginal gaps of restorations fabricated by two techniques [4, 43, 44]. The differences in marginal gaps may be attributed to the inherent disparities in fabrication processes, where milled zirconia benefits from a subtractive process using pre-sintered blocks, ensuring higher precision and minimal material shrinkage after sintering. In contrast, the 3D printing process, which involves layer-by-layer deposition, can introduce inaccuracies due to factors such as layer resolution, material shrinkage during sintering, and surface irregularities from the printing process [1]. Additionally, we emphasize that zirconia used in 3D printing may experience greater shrinkage during sintering compared to pre-sintered milling blanks, potentially resulting in larger marginal discrepancies if not properly compensated for during the design phase [1, 45]. Furthermore, we acknowledge that the post-processing steps for 3D-printed zirconia, which are typically more extensive compared to milled zirconia, could contribute to variability in the marginal fit [45].

The marginal gap increased within each group following the cementation process, the mean marginal gaps of milled zirconia crowns (group M) before and after cementation, were 20 μm, 22 μm, while the mean marginal gaps of 3D printed zirconia crowns (group P) were 41 μm and 42 μm respectively. Numerous studies comparing the marginal gap before and after cementation concluded that cement had a detrimental effect on the marginal gap, which was exacerbated following cementation [35, 45–47]. Even when applied in minimal amounts, the cement layer adds material between the tooth and the crown, which may cause the restoration to shift slightly away from the tooth’s margins, thereby increasing the marginal gap. This effect can also be influenced by the viscosity of the cement, as thicker cements tend to result in larger gaps.

The limitations of this study lie in its simulation period and environmental scope. Specifically, the study utilized 75,000 loading cycles to replicate six months of function, which may not fully capture the long-term effects on zirconia restorations. Future research should extend the aging period to provide a more comprehensive evaluation of long-term outcomes. Additionally, the study did not examine the impact of environmental factors such as saliva, beverages, or pH fluctuations, which are critical in simulating the oral environment. Investigating these variables in future studies will provide a deeper understanding of how zirconia crowns perform under more realistic conditions.

## Conclusions

Within the limitations of this in-vitro study, the following conclusions could be drawn:

1. 3D printed zirconia crowns showed higher marginal gap values compared to milled zirconia crowns, although both types remained within the clinically acceptable range.
2. The cementation process significantly impacted the marginal gap for both milled and 3D printed zirconia restorations, emphasizing the critical role of luting materials in crown marginal adaptation. The application methods and selection of the appropriate cement are vital in minimizing this effect.
3. While 3D printed zirconia has shown promising properties in research settings, Further clinical studies and regulatory evaluations are required to establish its long-term safety and efficacy to receive a widespread regulatory approval for routine intraoral use.

## Data Availability

No datasets were generated or analysed during the current study.

## Abbreviations

DLP: Digital Light Processing
Y-TZP: Yttria-stabilized Tetragonal Zirconia Polycrystal
CAD/CAM: Computer-Aided Design/Computer-Aided Manufacturing
AM: Additive Manufacturing
CNC: Computer Numerical Control
CEJ: Cementoenamel Junction
SPD: Sample Positioning Device
